# Evaluating effectiveness of screening house eaves as a potential intervention for reducing indoor vector densities and malaria prevalence in Nyabondo, western Kenya

**DOI:** 10.1186/s12936-020-03413-3

**Published:** 2020-09-19

**Authors:** Peter Njoroge Ng’ang’a, Collins Okoyo, Charles Mbogo, Clifford Maina Mutero

**Affiliations:** 1 International Centre of Insect Physiology and Ecology (ICIPE), PO Box 30772, Nairobi, Kenya; 2 Jomo Kenyatta University of Agriculture and Technology, School of Public Health, PO Box 62000, Nairobi, Kenya; 3 Eastern and Southern Africa Centre of International Parasite Control (ESACIPAC), Kenya Medical Research Institute (KEMRI), Nairobi, Kenya; 4 KEMRI-Wellcome Trust Research Programme, Public Health Unit, PO Box 43640 - 00100, Nairobi, Kenya; 5 University of Pretoria Institute for Sustainable Malaria Control (UP ISMC, School of Health Systems and Public Health, University of Pretoria, Private Bag X363, Pretoria, 0001 South Africa

**Keywords:** Anopheles *gambiae*, Eaves, Screening, Malaria, Prevalence, Mosquitoes, Vector control

## Abstract

**Background:**

Mosquito-proofing of houses using wire mesh screens is gaining greater recognition as a practical intervention for reducing exposure to malaria transmitting mosquitoes. Screening potentially protects all persons sleeping inside the house against transmission of mosquito-borne diseases indoors. The study assessed the effectiveness of house eaves screening in reducing indoor vector densities and malaria prevalence in Nyabondo, western Kenya.

**Methods:**

160 houses were selected for the study, with half of them randomly chosen for eaves screening with fibre-glass coated wire mesh (experimental group) and the other half left without screening (control group). Randomization was carried out by use of computer-generated list in permuted blocks of ten houses and 16 village blocks, with half of them allocated treatment in a ratio of 1:1. Cross-sectional baseline entomological and parasitological data were collected before eave screening. After baseline data collection, series of sampling of indoor adult mosquitoes were conducted once a month in each village using CDC light traps. Three cross-sectional malaria parasitological surveys were conducted at three month intervals after installation of the screens. The primary outcome measures were indoor *Anopheles* mosquito density and malaria parasite prevalence.

**Results:**

A total of 15,286 mosquitoes were collected over the two year period using CDC light traps in 160 houses distributed over 16 study villages (mean mosquitoes = 4.35, SD = 11.48). Of all mosquitoes collected, 2,872 (18.8%) were anophelines (2,869 *Anopheles gambiae *sensu lato, 1 *Anopheles funestus* and 2 other *Anopheles spp*). Overall, among *An. gambiae* collected, 92.6% were non-blood fed, 3.57% were blood fed and the remaining 0.47% were composed of gravid and half gravid females. More indoor adult mosquitoes were collected in the control than experimental arms of the study. Results from cross-sectional parasitological surveys showed that screened houses recorded relatively low malaria parasite prevalence rates compared to the control houses. Overall, malaria prevalence was 5.6% (95% CI: 4.2–7.5) n = 1,918, with baseline prevalence rate of 6.1% (95% CI: 3.9–9.4), n = 481 and 3^rd^ follow-up survey prevalence of 3.6% (95% CI: 2.0–6.8) n = 494. At all the three parasitological follow-up survey points, house screening significantly reduced the malaria prevalence by 100% (*p* < 0.001), 63.6% (*p* = 0.026), and 100% (*p* < 0.001) in the 1st, 2nd and 3rd follow-up surveys respectively.

**Conclusions:**

The study demonstrated that house eave screening has potential to reduce indoor vector densities and malaria prevalence in high transmission areas.

## Background

Malaria remains a major public health problem in Kenya, accounting for approximately 21% of outpatient consultations and 3–5% of hospital admissions each year [1]. The disease prevalence in the country varies by season and across geographic regions [2, 3]. Areas in the western and eastern parts of the country, respectively around Lake Victoria and at the coast, present the highest risk with children under five years and pregnant women being the most vulnerable to infection [4]. The Government of Kenya places a high priority on malaria prevention and control, with eventual malaria elimination listed as one of the strategic objectives of the Kenya Health Policy [1].

Through the Ministry of Health (MOH), the National Malaria Control Programme (NMCP) has implemented sound policies and evidence-based strategies in the fight against malaria. Key interventions include the provision of long-lasting insecticidal nets (LLINs), intermittent preventive treatment for pregnant women, and prompt diagnosis and effective treatment of all malaria cases with appropriate drugs, particularly artemisinin-based combination therapy [1, 4]. Other interventions include improving the capacity of health providers and strengthening the supply chain to deliver diagnostic tests and quality-assured medicines at all levels of the health system.

Vector control is among the widely recognized effective measures for prevention of malaria transmission and it constitutes a core strategy for malaria control in the African region [4, 5]. The most common vector control options are LLINs, and indoor residual spraying (IRS) using insecticides approved by the WHO Pesticide Evaluation Scheme (WHOPES) [6, 7]. The two insecticide-based methods although regarded as being among the key factors that contributed to 50–60% global reduction of malaria between 2000 and 2015 are faced with serious challenges, most notably widespread insecticide resistance among mosquito populations [8–11]. The World Health Organization’s response to these perennial challenges includes the promotion of integrated vector management (IVM), involving combining of existing primary interventions of LLINs and IRS with complementary methods, such as house improvements and other environmental management measures [12–15].

Historically, destruction of vector breeding habitat, application of IRS using insecticides, such as Dichlorodiphenyltrichloroethane (DDT) and improvement of housing structures have been shown to reduce indoor vector densities and malaria transmission [16–18]. The interventions have in the past significantly contributed to suppression or elimination of malaria and yellow fever. In the early twentieth century, screening and improvements in housing helped bring about marked reductions in malaria across different settings, most notably in the USA [16, 19].

Mosquito-proofing of houses is particularly important because the main *Anopheles* species of mosquitoes transmitting malaria in Africa bite between dusk and dawn when people are asleep indoors. They enter human dwellings through open windows, doors and eaves, largely attracted by human odour [20]. Open eaves are significant entry points into houses for malaria vector species and are recognized as a risk factor for malaria transmission in endemic regions [21]. Therefore, housing improvement incorporating screening as a physical barrier against mosquito entry is potentially an effective mechanism of reducing malaria transmission. This fact was also recognized by the Roll Back Malaria (RBM) Vector Working Group and its partners in their November 2015 consensus statement on housing and Malaria [22]. The statement argues that while traditional preventive measures, such as treated mosquito nets and insecticide spraying has greatly contributed in lowering malaria incidence and deaths, they need to be complemented by other measures of malaria control and elimination, such as house screening because it covers and protects all individuals in a house equally [20, 22].

Perhaps the most obvious way of preventing mosquitoes from entering a house is to screen the entry points with a mesh. A trial of house screening in The Gambia showed that screening led to a reduction in the number of mosquitoes entering houses and in the prevalence of anaemia in children [23]. Among the potential advantages of house screening is the equity with which it protects all members of the household at all times while indoors [24, 25], unlike LLINs which primarily give protection to those with a net during sleeping hours only. An additional benefit of house screening is its potential for integration with other vector control and disease management interventions, offering protection from both malaria and other vector borne diseases such as lymphatic filariasis [26, 27].

Recent systematic reviews and meta-analysis [21, 24, 25] have underscored the need for further studies on housing improvements in Africa and other parts of the tropics. Additional research can give clarity regarding situations in which house screening is an effective malaria intervention, since certain studies have shown that screening can be effective in preventing mosquito entry into houses, but with little evidence as to how much it can reduce malaria infection [28].

The systematic reviews have also highlighted the absence of data from many geographical regions, lack of enough intervention studies and the high risk of bias within and across studies [21, 25, 27]. Progress towards building the required body of scientific evidence includes a randomized controlled trial (RCT), conducted in the Gambia, which evaluated a house screening intervention against malaria, epidemiological outcomes and social acceptability [23, 29].

A recent survey on community perceptions and knowledge regarding the protective nature of house screening in the study area showed that more than 85% of household owners considered screening useful, although majority of houses in the area were not screened [29]. Reasons given for not screening the houses included perceptions that it was costly, and also lack of awareness regarding its effectiveness in protecting against malaria. The objective of this study was to evaluate the effectiveness of house-eaves screening in reducing both indoor resting malaria vector densities and malaria prevalence.

## Methods

### Study area

**Fig. 1:**
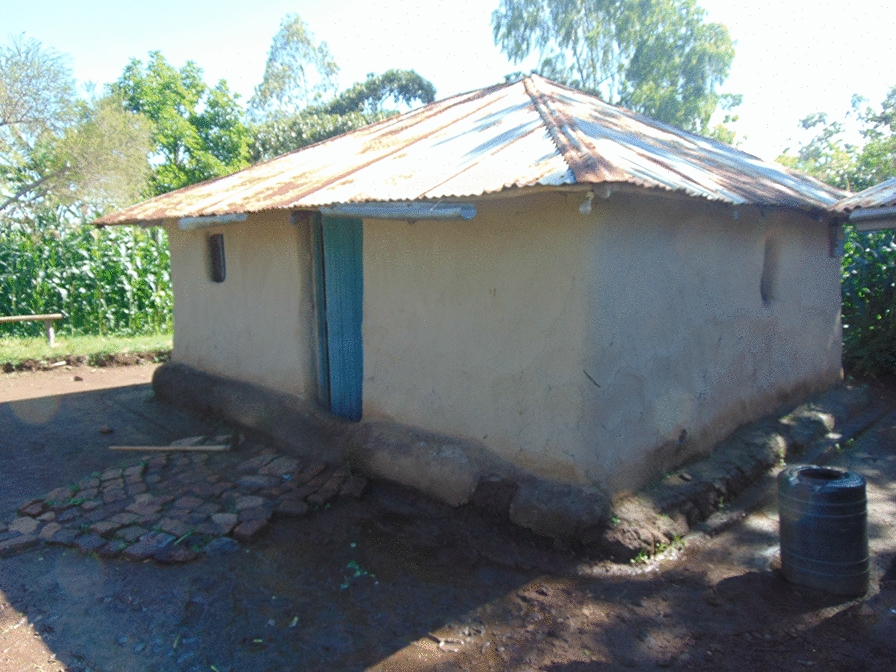
An ordinary local house in the study area showing the wall and the roofing type

The study was carried out in Nyabondo, a plateau area located in Upper Nyakach sub-county of Kisumu County, about 30 km North-East of Lake Victoria. Nyabondo lies between an altitude of 1520 m and 1658 m above sea level, and 0° 23′ 0 S and 34° 58′ 60 E. The area is host to an estimated 34,000 people with a high population density of nearly 460 persons per square kilometre (km) [31]. The community largely depends on brick making as the main economic activity with small-scale mixed farming activities such as crop/fish farming and livestock keeping. Agricultural activities are dominated by crops such as maize, cassava, sorghum and sweet potatoes [15, 32]. Malaria is endemic in this region and its perennial transmission is mainly determined by rainfall, temperature and humidity [4, 33]. The reported average malaria parasite prevalence in the human population was about 27% in 2015 [1, 4, 33]. Previous entomological surveys in Nyabondo found that larval *Anopheles* mosquitoes bred in both temporary and permanent aquatic habitats with *Anopheles arabiensis* being the main malaria vector species (99.3%), followed by *Anopheles gambiae* (0.7%) [32, 34, 35]. In 2015/2016, the overall poverty headcount rate for individuals at the national level was 36.1%. The highest overall poverty incidence nationally was reported in rural areas, where 40.1% of the residents were considered as overall poor, compared to 27.5% in peri-urban areas and 29.4% in core urban areas [36]. Being a rural setup, the overall poverty incidence in Nyabondo was approximated at 40.1% [36]. The majority of houses in Nyabondo are made up of local design, constructed with iron sheets roofs, mud walls and a plaster finishing walls (Fig. 1) made by mixing ash, mud and cow dung [30].

### Study design

A cluster randomized study design was implemented to study the effect of house eaves screening [37, 38]. An initial household enumeration exercise was conducted and randomization carried out by use of computer-generated list, in permuted blocks of ten houses and 16 village blocks in the study site, with 80 houses in each of the two study arms (control and intervention) for both entomological and parasitological assessment. In each village, there were 10 houses randomly selected for the study with half of them allocated treatment in a ratio of 1:1 between the control and treatment groups. Considering the high average malaria prevalence (27%) normally reported in Nyabondo, and since the area is not vast geographically, being as it is within a sub-county, it was assumed that the population sampled from a total of 160 households would constitute a large enough sample capable of showing valid statistical difference in the measurements being taken.

### Entomological surveys

Sampling of adult mosquitoes was conducted from January 2017 to November 2018. The sampling was spread out over a period of 16 days each month, with all the ten houses in a particular village being sampled in one night. Sampling was conducted using CDC light traps with one light trap set up in an occupied bedroom per house and left to run for 12 h overnight between 19:00hours and 07:00hours. Mosquitoes collected in the morning were killed using chloroform and morphologically identified in the field station as either belonging to anopheline or culicine group [39, 40]. Subsamples of anophelines were also identified morphologically to species and sex and further separated by physiological state as either unfed and blood fed using keys of Gillies and De Meillon [40]. Adult mosquito collections were done simultaneously for two years in both intervention and control houses and were scheduled so as to span both dry and wet seasons. The number of female indoor adult *An. gambiae *sensu lato (*s.l.*) vectors collected per trap night were used as a primary outcome for assessing the efficacy of eave screening in reducing indoor malaria vector densities.

### Malaria prevalence surveys

Cross-sectional household malaria parasitological surveys were conducted after every three months from September 2017 to November 2018. Testing was done by Rapid Diagnostic Tests (RDTs), using SD-Bioline malaria antigen P.f® test as recommended by the NMCP in Kenya [41]. All members of the household were tested for the presence of malaria parasite using RDT [42] for four consecutive times (i.e. in every three months), this included one baseline survey in September 2017. The tests were performed by trained staff from the Ministry of Health following the manufacturer instructions [41, 42]. Individual socio-demographic information of the household members were collected in addition to the house characteristics. Consent to participate in this study was requested from the participants during the study. Individuals were asked whether they had taken any anti-malaria medication prior to the survey day. Participants found to be positive for malaria parasites were treated by Ministry of Health staff according to World Health Organization (WHO) and National Guidelines for the Diagnosis, Treatment and Prevention of Malaria in Kenya [41–43]. Malaria parasite rate was used as the secondary endpoint for assessing the efficacy of eave screening at household level.

### Eave screening procedure

**Fig. 2:**
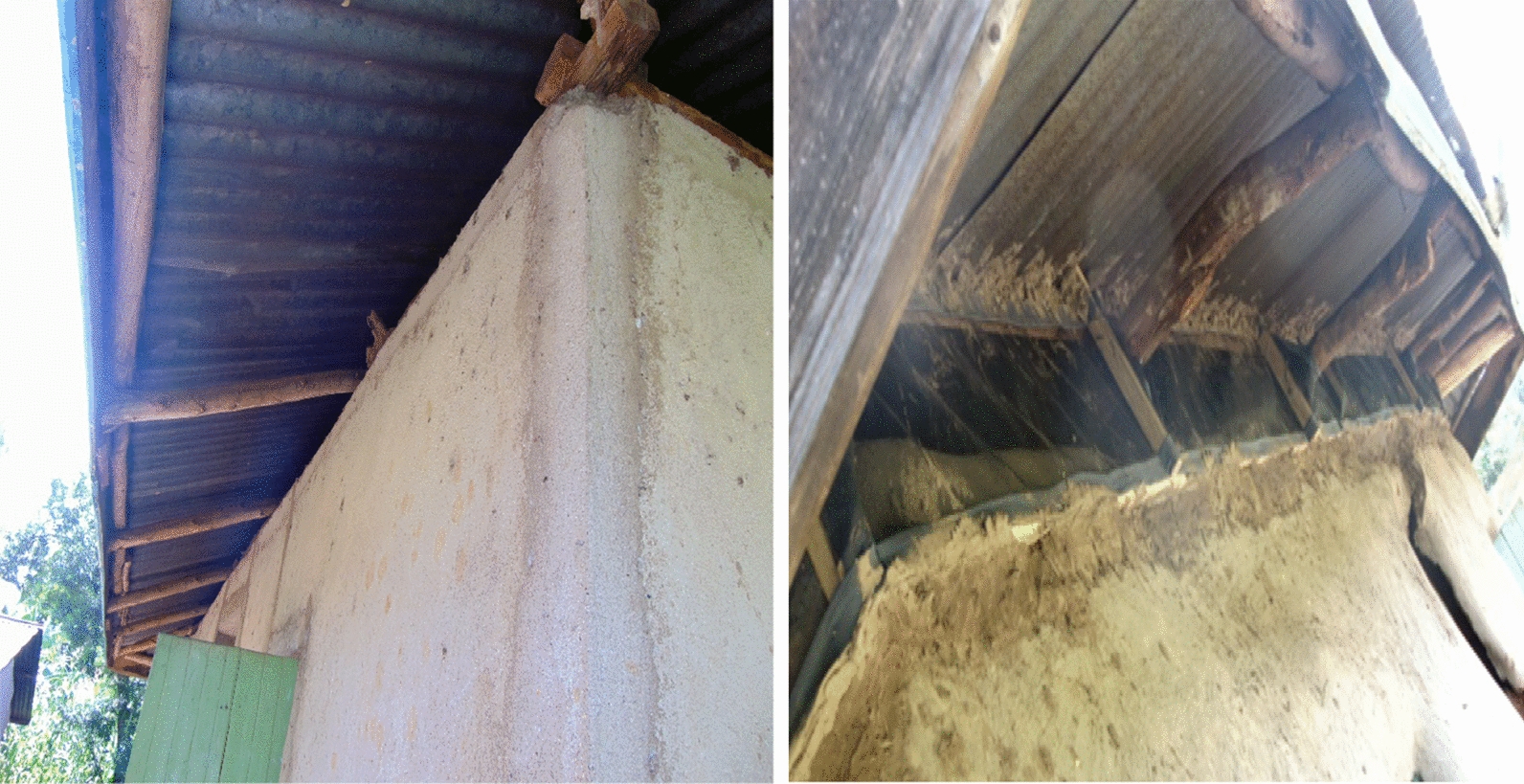
An ordinary house showing unscreened open eave on the left and a similar house on the right fitted with grey polyester netting material [screened eave]

After randomization and getting consent from household owners, the houses in the category to be screened were fitted with grey coloured fibre-glass coated wire mesh, designed to tightly and firmly fit onto pre-measured eave openings. Prior to fixing the screen in November–December 2017, an elastic cloth lining was sewn onto its edges by a tailor hired from the community. The lining is what was used to fix the screen on a wooden framework in the house eaves using one inch nails (2.54 cm) as a harness (Fig. 2). The screening work and roll-out roster was undertaken by project staff together with local youth, trained by an experienced consultant who had previous experience with house screening in other similar areas. During the screening activities, household occupants were encouraged to close windows and doors early in the evening in order to reduce mosquito entry into houses. Both Informed, verbal and written consent were sought from the head of each household before screens were installed into houses.

### Health facility data collection

Data on laboratory confirmed malaria cases was collected from the area mission health facility in Nyabondo and recorded every month during 2017–2018 period. A data collection form was developed and used to capture this information. Malaria case detection form was used to summarize cases based on age, gender and home locality of the patient. Health-seeking in the area is primarily from MOH and faith-based facilities such as the mission health facility in Nyabondo since they are either free or charge minimal user fees for their services.

### Rainfall data

Rainfall data was obtained from the local meteorological department situated within the study area. Monthly average rainfall for the two years was computed and used in the analysis.

### Data management and statistical analysis

Data collected was counter-checked for accuracy and verified before double entry into computer MS Excel spreadsheet. The double entry was done by an independent person, who also checked for data entry errors. Adult mosquito relative density was defined as the number of female adult *Anopheles* mosquitoes per house per night. The 95% confidence intervals (CIs) for the mean adult mosquito relative density were estimated using negative binomial regression model adjusted for household clusters. Mean mosquito population densities and the relative abundance of different vector species were compared between 2017 and 2018. The effect of house screening intervention on adult mosquito density was estimated using generalized estimating equations (GEE) [44], allowing for within-subject correlation using robust variance estimator to calculate standard errors (SEs). From the GEE model, we reported the incidence rate ratios (IRRs), the control arm (unscreened houses) was used as the reference against the experimental (screened houses).

For malaria epidemiological survey, *Plasmodium falciparum* infection was defined as a positive rapid diagnostic test (RDT) result. Proportion of individuals infected with malaria was calculated and the 95% confidence intervals (CIs) estimated using binomial regression model that accounted for household clusters. The impact of house screening on infection prevalence was calculated and odds ratios (ORs) estimated using multilevel mixed effects logistic regression model while accounting for household clusters. All statistical analyses were performed using STATA version 14.1.

## Results

### Characteristics of study population

In total, 160 households participated in both entomological and parasitological surveys. Around thirty two percent (31.9%) of the study household heads/spouses had completed primary school education and 16.9% had dropped out at secondary schools. In terms of occupation, majority of the household (91.9%) were farmers, 5% in self business and 1.3% were either in brick making or not in any formal employment (Table 1). Around fifty two percent (51.87%) of the households had 4–6 occupants with a median of 4 and a range of 1–8.

**Table 1:** Characteristics of study population

	Variable	Frequency	%
Education of HH head	Primary school (Not completed)	30	18.8
	Primary school (Completed)	51	31.9
	Secondary school (Not completed)	27	16.9
	Secondary school (Completed)	25	15.6
	University/college	15	9.4
	Informal education	12	7.5
	Total households	160	100
Main occupation of HH head	Student	1	0.6
	Farming	147	91.9
	Self-business	8	5
	Unemployed	2	1.3
	Brick making	2	1.3
	Total households	160	100

### Indoor adult mosquito collections

Overall, 15,286 mosquitoes were collected over the two years using CDC light trap in 160 houses distributed over 16 study villages (mean = 4.35, SD = 11.48). Of all mosquitoes collected, 2,872 (18.8%) were anophelines (2,869 *An. gambiae s.l*., 1 *Anopheles funestus* and 2 other *Anopheles* spp), and 12,414 (81.2%) were culicines. Of the total 12,414 culicines collected in the two years, 20.6% were collected from screened houses compared to 79.4% collected from unscreened (control) houses respectively. There was overall low number of *Anopheles* mosquitoes collected indoor in experimental houses compared to control houses during the sampling period despite non-significant difference in the mean number of mosquitoes (Additional file 1).

A total of 14,952 mosquitoes were unfed (2,749 *An. gambiae*, 0 *An. funestus*, and 12,203 culicines), 311 mosquitoes were fed (106 *An. gambiae*, 0 *An. funestus*, and 205 culicines), 6 were half gravid (6 *An. gambiae*, 0 *An. funestus*, and 0 culicines), and 15 were gravid (8 *An. gambiae*, 1 *An. funestus*, and 6 culicines). Overall, among *An. gambiae* collected, 92.6% were non-blood fed, 3.6% were blood fed and the remaining 0.5% were composed of gravid and half gravid females. However, irrespective of the species, more unfed, fed, half gravid and gravid mosquitoes were collected in the control than experimental arms of the study (Table 2).

**Table 2:** Number of indoor adult mosquitoes collected in the study area, separated by species and physiological state

Mosquito species	Unfed N (mean; SD)	Fed N (mean; SD)	Half gravid N (mean; SD)	Gravid N (mean; SD)
Control				
* An. gambiae*	2208 (0.78; SD = 3.13)	103 (0.04; SD = 0.90)	6 (0; SD = 0.11)	8 (0; SD = 0.09)
* An. funestus*	0	0	0	1 (0; SD = 0.02)
Other anopheles (unidentified)	0	0	0	0
Culicines	9658 (3.40; SD = 8.88)	197 (0.07; SD = 1.01)	0	3 (0; SD = 0.03)
Experimental				
* An. gambiae*	541 (0.80; SD = 3.15)	3 (0; SD = 0.09)	0	0
* An. funestus*	0	0	0	0
Other Anopheles	0	0	0	0
Culicines	2545 (3.75; SD = 12.82)	8 (0.01; SD = 0.15)	0	3 (0; SD = 0.09)
Overall				
* An. gambiae*	2749 (0.78; SD = 3.14)	106 (0.03; SD = 0.81)	6 (0; SD = 0.10)	8 (0; SD = 0.08)
* An. funestus*	0	0	0	1 (0; SD = 0.02)
Other *Anopheles* spp	0	0	0	0
Culicines	12,203 (3.47; SD = 9.76)	205 (0.06; SD = 0.91)	0	6 (0; SD = 0.05)

**Fig. 3:**
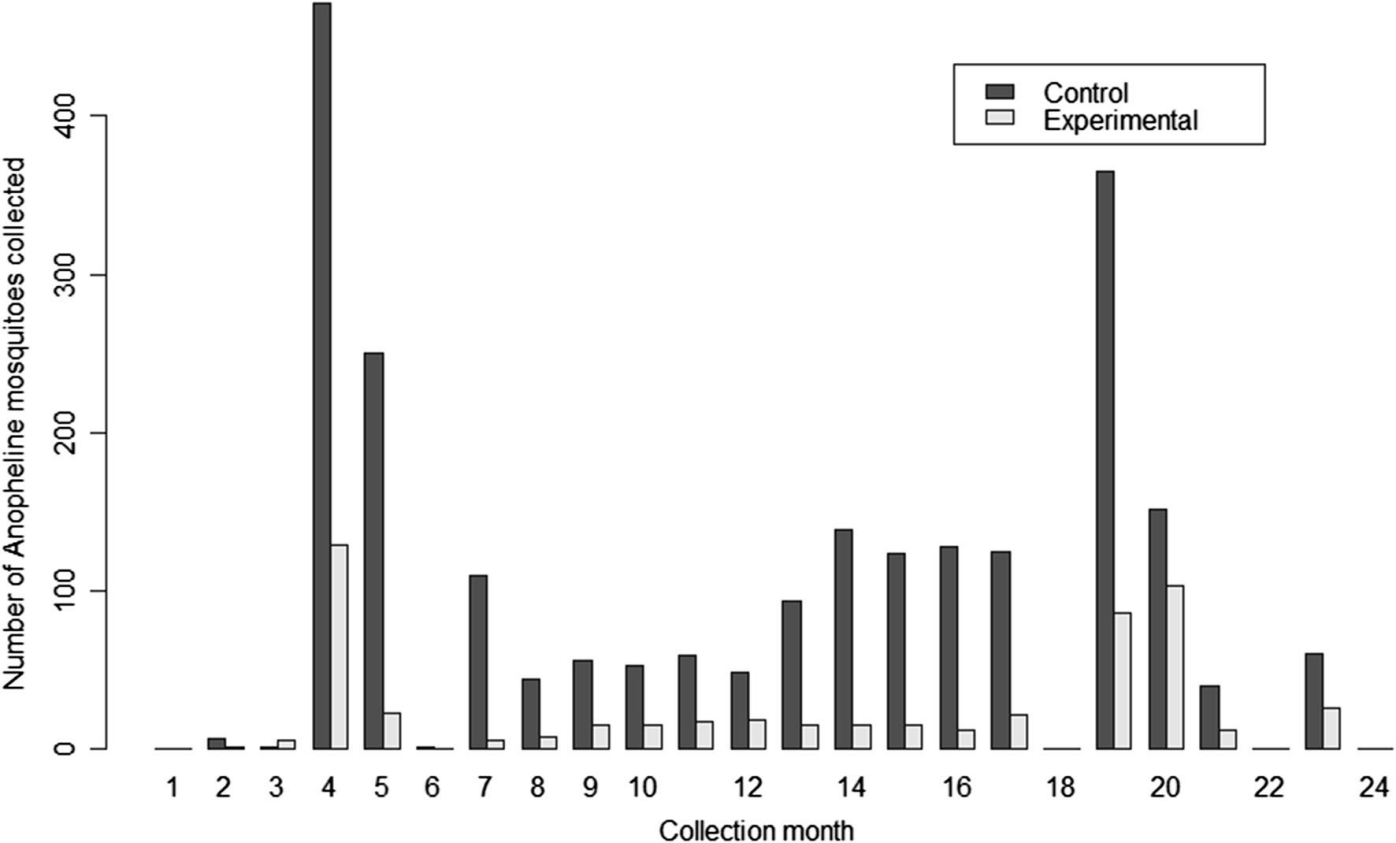
Time series trend and comparison of the monthly collections of the anopheline mosquitoes for two years

Figure 3 gives the time series trend and comparison of the monthly collections of the anopheline mosquitoes. Baseline collections were done during the first nine months (i.e. January to September of 2017), and it cumulatively showed higher number of mosquitoes especially during the months of April and May. Further, the follow-up collection periods showed a gradual increase in the number of mosquitoes, which peaked around the months of July and August of the following year, before sudden decline to very low levels. Importantly, at most months, the total number of mosquitoes in the experimental group were lower than those in the control group.

### Impact of house screening on malaria prevalence

Overall, malaria prevalence was 5.6% (95% CI: 4.2–7.5) n = 1,918, with baseline prevalence of 6.1% (95% CI: 3.9–9.4), n = 481 and 3^rd^ follow-up survey prevalence of 3.6% (95% CI: 2.0–6.8) n = 494. At all the three follow-up survey points, house screening significantly reduced the malaria prevalence by 100% (*p* < 0.001), 63.6% (*p* = 0.026), and 100% (*p* < 0.001) in the 1st, 2nd and 3rd follow-ups respectively**.** Analysis of the prevalence when baseline survey was not taken into account is also given in Table 3, and overall, there was no much noticeable difference in results with and without baseline survey. When baseline survey was not taken into account, greater impact of house screening in reducing malaria prevalence was observed at 80% (*p* = 0.005) (Table 3).

**Table 3:** House eaves screening impact on malaria parasite prevalence rate in the study area

Characteristic	Baseline survey	1st follow-up	2nd follow-up	3rd follow-up	Overall	
					With baseline	Without baseline
Malaria parasite prevalence, % (95% CI)*						
Control	5.2 (3.4–8.1)	6.9 (5.0–9.4)	10.1 (6.6–15.3)	4.1 (2.2–7.9)	6.3 (4.8–8.3)	6.7 (4.9–9.2)
Experimental	10 (4.5–22.2)	0	3.7 (1.3–10.7)	0	3.0 (1.5–6.0)	1.4 (0.4–4.5)
Risk reduction, % (*p *value)	*Increase*	100% (*p* < 0.001)	63.6% (*p* = 0.026)	100% (*p* < 0.001)	52.4% (*p* = 0.019)	79.1% (*p* = 0.006)
Total prevalence	6.1 (3.9–9.4)	4.6 (3.5–5.9)	8.2 (5.1–13.2)	3.6 (2.0–6.8)	5.6 (4.2–7.5)	5.4 (3.9–7.5)
*House screening impact on malaria parasite prevalence, OR (95% CI), p value*^@^						
Control	Reference					
Experimental	2.01 (0.88–4.57), *p* = 0.096	0, *p* < 0.001	0.35 (0.14–0.85), *p* = 0.021	0, *p* < 0.001	0.46 (0.24–0.87), *p* = 0.017	0.20 (0.06–0.61), *p* = 0.005

*Malaria parasite prevalence was calculated and 95% confidence intervals estimated using binomial logistic regression model while accounting for house clustering

^@^The impact of house screening on malaria prevalence was calculated and odds ratios (ORs) estimated using multilevel mixed effects logistic regression model while accounting for house clustering

**Fig. 4:**
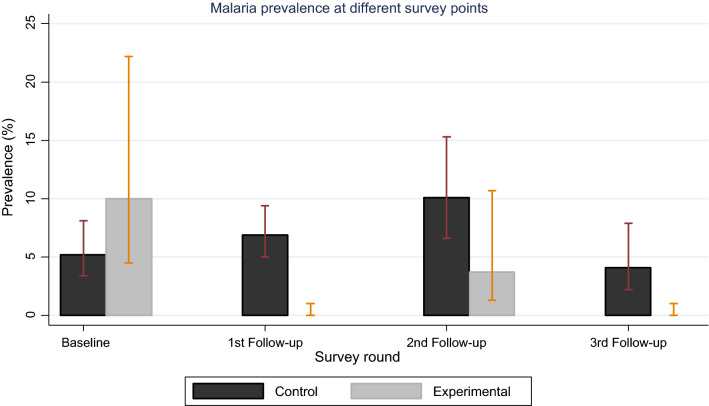
Comparison of malaria prevalence in both control and experimental groups at different survey points

Figure 4 compares the malaria prevalence in both control and experimental groups at different survey points. Overall, house screening significantly reduced malaria prevalence by 54% (OR = 0.46, 95% CI: 0.24–0.87, *p* = 0.017), there were also significant impacts observed at each of the three follow-up survey points.

### Vector densities and malaria cases in relation to rainfall pattern

**Fig. 5:**
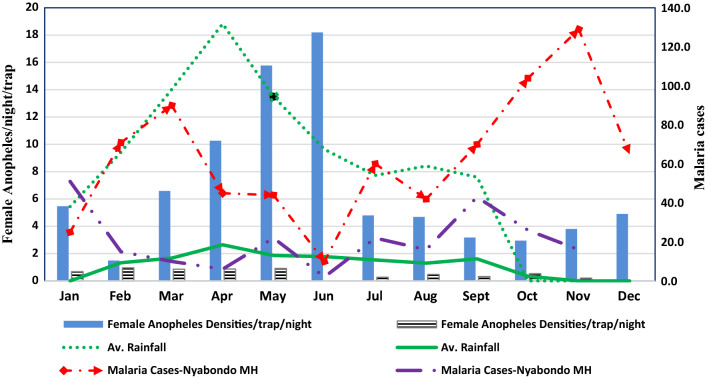
Female *Anopheles* mosquito densities/night/trap in relation to rainfall and malaria cases in the study area

Female *Anopheles* mosquitoes collected indoors per trap/night reduced significantly from 2017 to 2018. In 2017, there was relatively high numbers of captured indoor malaria vectors that corresponded with a similar increase in recorded average rainfall especially for the months of March-June. The same year and months also recorded a similar increase in recorded malaria cases in the nearby Nyabondo Mission Hospital compared to year 2018 (Fig. 5). The high numbers of recorded malaria cases at the hospital for the months of September–November 2017 was attributed to national wide strike of nurses and clinicians working in government health facilities in the country. It was observed when the local government health facilities were not functioning, more local people sought health services from the mission hospital which also doubled as a referral facility serving people from other surrounding sub-counties. Comparatively, the year 2018 recorded reduced amount of rainfall, less numbers of malaria vectors.

## Discussion

Housing structure has been shown to be one of the factors influencing indoor vector densities and malaria transmission [21, 24, 25]. Results from this study demonstrated that screening of house eaves has the potential of reducing the number of mosquitoes collected indoors and also malaria prevalence in a rural African setting of high malaria endemicity. These observations and those from prior assessment of community perception of house screening at the same study site [30] challenged a common notion elsewhere that screening interventions would be expensive, impractical and without benefit in rural African settings with houses that are constructed of mud walls and grass thatched roofs [45]. Poorly constructed houses with unscreened eave gaps would intuitively have high human-vector exposure compared to screened houses, resulting in a correspondingly higher risk of malaria transmission.

From this study, there was overall total reduction of *Anopheles* mosquitoes collected indoor in experimental houses compared to control houses during the study period. Similar observations have been made in the Gambia and Ethiopia where houses fitted with ceiling, door and window screens recorded a decrease in indoor resting mosquitoes [23, 47–49]. Other studies have demonstrated that major malaria vectors are predominantly endophagic, nocturnal and mainly enter houses through the eaves and more than 80% of malaria transmission occurs indoors, primarily at night or in the later part of the night [49, 50]. Night time is, therefore, when most people are bitten and infected with malaria or certain other mosquito-borne pathogens such as those that cause lymphatic filariasis, transmitted by both anopheline and culicine mosquitoes. Of the total culicines collected in our study area, a large proportion was from the unscreened (control) houses. The importance of eaves as the preferred entry point for *Anopheles* mosquitoes has been recognized by the WHO since 1997 [18]. Thus, screening the gaps has the potential of lowering the occurrence of malaria, where mosquitoes usually feed on people indoors [48, 49, 51]. In a related survey in Baringo, Kenya, houses with closed eaves had a low mean number of mosquitoes compared to houses with open eaves [52]. Elsewhere in East Africa, poor housing construction was associated with increased malaria incidence in a cohort of young Ugandan children [53]. Also, in western Kenya, Atieli et al. 2009 demonstrated that house design modification by inclusion of a ceiling can reduce mosquito densities considerably [54].

In all the three follow-up survey points, house screening significantly reduced malaria prevalence. Screened houses recorded relatively low malaria prevalence rates in the study area compared to the control houses for the whole period. However, during the third prevalence survey (second follow up) that was conducted in July–August 2018, both the intervention and control arms experienced an increase in prevalence rates. This could speculatively be partly attributed to less usage of personal protection measures during the hot seasons of the year when the rains subsided in the study area (Fig. 5). The potential effect of house screening in reducing malaria prevalence and incidences has been demonstrated in other related studies in Africa [21, 23, 46, 47, 51, 53]. A randomized trial in the Gambia also showed that screening was effective in reducing the level of anaemia in children [23] and in Dar es Salaam Tanzania, house screening contributed to a reduction in malaria transmission [46, 55].

Eave screening is most likely to be successful and accepted in areas where a large reduction in indoor biting is experienced by the occupants and also in households where people prefer not to use bed nets, or have stopped using them. Another potential advantage of house improvements and screening is the equity with which it protects all members of the household at all times while indoors, unlike LLINs which primarily give protection to those with a net during sleeping hours only. Nevertheless, eaves screening has also been found to affect compliance to LLNs use in areas where household members perceive that house screening offers them sufficient protection against mosquito bites [29]. Perhaps the greatest benefit to house screening would be its potential for integration with other vector control strategies like larval source management and bed net use. There is evidence that interventions which impede mosquito entry to houses could also protect inhabitants against vector-borne diseases such as leishmaniasis, dengue fever, yellow fever, zika virus, chikungunya, and lymphatic filariasis [26, 27].

Ecological and climatic factors have been reported to be important drivers of malaria transmission in sub-Saharan Africa [56–58]. Earlier studies in Western Kenya have attributed meteorological/climatic factors with the occurrence of *P. falciparum* malaria parasites, mosquito vector densities and risk of malaria transmission [56, 58, 59]. Vector availability, biting rates and parasite development are all influenced by climatic conditions as it relates to various *Plasmodium* species [56]. Factors such as temperature, humidity and rainfall directly impact the lifecycles of both vector and parasite [58]. Rainfall aids in accumulation of stagnant water, hence making the environment ideal for mosquito breeding sites, whereas, higher temperatures are associated with accelerated *Plasmodium* incubation period within mosquitoes [57, 58].

## Limitations of the study

Doors and windows were not fitted with the screens. There is, therefore, a possibility that these could serve as mosquito entry points if left open during the night, thereby leading to a distortion of the results. However keeping doors and windows open at night is not common in the study area due to fear of theft. Through community engagements, household owners were also encouraged to close the windows and doors early in the evenings for purposes of the experiment. The imbalances between the arms could have been partly due to randomization error and use of CDC light traps in sampling indoor adult mosquitoes, which is associated with efficiency issues in estimating indoor vector densities at household level [60, 61]. Another limitation related to the number of malaria cases recorded at the mission hospital. It was not possible to determine the impact of the house screening in the community based on health facility data due to confounding factors such as climatic factors like rainfall, drought periods, strike action by MOH staff and the fact that the mission hospital was not the sole health service provider in the study area. The authors recommend further research on protective efficacy of house screening (doors, windows and eaves) before scaling up the intervention to the wider community in the study area. Among the households involved in the study accepted the intervention though further research is needed to assess the durability, cost-effectiveness and social acceptability of the intervention over time.

## Conclusion

House eave screening is an effective and promising strategy for reducing indoor vector densities and malaria prevalence in high transmission rural areas. It also has the potential of being integrated with other control strategies in malaria endemic areas of Sub Saharan Africa.

## Supplementary information

**Additional file 1.** Number of indoor adult mosquitoes collected in Nyabondo for two years, separated by species and the study arm.

## Data Availability

The data and materials used for this study are available from the corresponding author upon request.

## Abbreviations

CDC: Center for Disease Control
CIs: Confidence Intervals
DDT: Dichloro-Diphenyl-Trichloroethane
GEE: Generalized Estimating Equations
ICIPE: International Centre of Insect Physiology and Ecology
IRRs: Incidence Rate Ratios
IRS: Indoor Residual Spraying
ITNs: Insecticide-Treated Nets
IVM: Integrated Vector Management
KEMRI: Kenya Medical Research Institute
LLINs: Long-Lasting Insecticide-Treated Nets
MOH: Ministry of Health
NMCP: Malaria Control Programme
OR: Odds Ratio
RBM: Roll Back Malaria
RCT: Randomized Controlled Trial
RDTs: Rapid Diagnostic Tests
SD: Standard Deviation
SE: Standard Error
SERU: Scientific and Ethic Review Unit
WHO: World Health Organization
WHOPES: WHO Pesticide Evaluation Scheme
