# Supplementary material for: Evaluating effectiveness of screening house eaves as a potential intervention for reducing indoor vector densities and malaria prevalence in Nyabondo, western Kenya
Source: Malar J. 2020 Sep 19;19:341. doi: 10.1186/s12936-020-03413-3 (PMC7501660; doi:10.1186/s12936-020-03413-3)
Supplement: Supplementary file 1 — Additional file 1. Number of indoor adult mosquitoes collected in Nyabondo for two years, separated by species and the study arm. [file 12936_2020_3413_MOESM1_ESM.docx]

Supplementary material 1: Number of indoor adult mosquitoes collected in Nyabondo for two years, separated by species and the study arm.

| **Study arm** | ***An. gambiae***  **N (mean; SD)** | ***An. funestus***  **N (mean; SD)** | **Other anopheles**  **N (mean; SD)** | **Culicines**  **N (mean; SD)** | **Total**  **N (mean; SD)** |
| --- | --- | --- | --- | --- | --- |
| Control | 2,325 (0.82; SD=3.77) | 1 (0; SD=0.02) | 2 (0; SD=0.03) | 9,858 (3.47; SD=9.11) | 12,186 (4.29; SD=10.84) |
| Experimental | 544 (0.80; SD=3.17) | 0 (0; SD=0) | 0 (0; SD=0) | 2,556 (3.77; SD=12.85) | 3,100 (4.57; SD=13.83) |
| Diff (t-test; p-value) | t=-0.108, p=0.914 | t=-0.489, p=0.625 | t=-0.691, p=0.489 | t=0.698, p=0.486 | t=0.567, p=0.571 |
| Overall | 2,869 (0.82; SD=3.66) | 1 (0; SD=0.02) | 2 (0; SD=0.02) | 12,414 (3.53; SD=9.94) | 15,286 (4.35; SD=11.48) |
| Diff: P-value for the statistical difference in the mean number of mosquito collections in the control and experimental groups was calculated using student t-test at 95% confidence interval. | | | | | |
